# Proposed New Regulations as to Medical Benefit

**Published:** 1913-11-29

**Authors:** 


### NATIONAL INSURANCE ACT DEVELOPMENTS.

### Proposed New Regulations as to Medical Benefit.

The latest important publication made by the Insurance Commissioners is the text of the regulations which they propose to issue to govern the administration of medical benefit. These regulations have not yet been definitely adopted, and the publication of the draft is designed to allow an opportunity for their discussion by interested parties. It is understood that the draft has already been fully discussed by the Advisory Committee, and it is clear that great care has been expended in its consideration. The time for further discussion is short, as it is proposed that the new regulations shall become operative as from January 12 next, and it will, of course, be necessary for the Insurance Commissioners to signify their authority to them some weeks in advance of that date.

The draft now submitted is naturally based to a large extent on the regulations at present in force, but it condenses into a single code several separate and distinct existing orders, and on this account alone it is to be welcomed as tending to facility of reference. New regulations were called for as the result of experience in working under the present system, particularly in regard to the distribution of the whole of the funds available for medical benefit. They were also required on account of the extension of the right to such benefit conferred by the Amendment Act passed this year.

The regulations are arranged under eight sections according to the nature of the subject to which they relate. This enables ready reference to be made to any particular regulation required, and is of importance seeing that there are no less than eighty-seven clauses, to which are added seven comprehensive schedules.

### Arrangements for Medical Benefit.

The first operative section deals with the arrangements for the provision of medical benefit. It will be remembered that medical benefit is not administered by the approved societies but by the Insurance Committees. But the Insurance Committees are not in direct touch with the insured persons apart from the comparatively small class of deposit contributors. It is therefore necessary that the Insurance Committees shall be furnished with lists of the names and addresses of the insured persons whom they are under obligation to supply with medical treatment and attendance. The Insurance Committees were placed in possession of such information at the inception of the scheme in January last by means of cards, which were designated "Index Slips." The compilation of this record, extending as it did to nearly fourteen million insured persons, was no mean task. In view of the constant removals of residence of the industrial population and to changes in membership of the societies, due to deaths, marriages, and transfers of existing members and the introduction of new members, the lists of insured persons are in a constant state of fluctuation. The approved societies have been under obligation therefore to keep the Insurance Committees regularly and punctually informed of all such alterations. It is somewhat astonishing, in the circumstances, that the proposed new regulations should require the approved societies to furnish, as soon as may be, lists of the names and addresses of their members to the appropriate Insurance Committees. If this refers to complete new lists it would seem that the approved societies have good reason to object to the amount of work thus thrust upon them. It may perhaps be the intention that societies which have already supplied the Insurance Committees with index slips and have kept the record up to date shall be exempt from such complete revision of their lists of members, but this is not made clear in the draft regulations so far as we can see. Beside the list of insured persons—to be known as "the Register"—each Insurance Committee is required to compile a list—called the medical list, or panel—of the practitioners who have entered into agreements with the Committee, and this list is to be published and available for inspection by insured persons at its offices and such other places as it may think fit. The conditions of service contained in the agreements between the Committees and the medical practitioners are, in all essential particulars, the same as those already existing. Provision is made for the revision of the terms of service should an Insurance Committee desire to make any alteration.

In like manner the list of persons supplying drugs and appliances is to be revised, kept up to date, and published in a form available for reference by insured persons. The conditions for inclusion in this list are not materially altered, but an important alteration is indicated in that it is to be made incumbent upon each Insurance Committee to constitute a special Pharmaceutical Service Sub-Committee to deal with complaints in respect of the quality of any drugs or appliances supplied, or in respect of any failure to supply drugs or appliances within a reasonable space of time. Insurance Committees are to be empowered, as formerly, to approve from time to time, with the sanction of the Insurance Commissioners, certain institutions for the provision of medical benefit when such institutions were in existence on the passing of the Act of 1911. The Insurance Committees are also to be given power, if they think fit, to impose an income limit and to require all persons in receipt of an income above such limit to make their own arrangements for receiving medical benefit. The Commissioners also permit the Committees to allow any insured persons, whether individually or collectively, in lieu of receiving medical benefit under arrangements made by the Committee, to make their own arrangements for receiving treatment. It is to be noted that this permission is, as formerly, at the option of each Insurance Committee, and there is no indication at present that these bodies are inclined to look with favour on such individual arrangements.

### Methods of Obtaining Medical Benefit.

In the next section the regulations deal with the methods of obtaining medical benefit. There are three methods—namely, treatment by a practitioner on the panel, or through an approved institution, or by individual arrangement. The foremost place is given to treatment under the panel system, which is still intended to be the normal method. One point that should be noted is that if an insured person, who is entitled to receive treatment under this method, finds difficulty in being accepted as a patient by any doctor on the panel the Insurance Committee is to make arrangements whereby the Panel Committee will assign him to a practitioner.

Since the pink medical benefit tickets, which were supplied to members by the approved societies in January last, were discontinued, we understand some difficulty has been experienced by the doctors in ascertaining whether insured persons coming to them were entitled to be entered on their books as insured patients. This difficulty is to be overcome by the issue of a list to each panel doctor of the persons who have been accepted by or assigned to him as insured patients.

Regulations are also put forward to meet the case of transfers from one doctor to another and to provide for those who remove from the area of one Committee to that of another. There seems to be little to object to in any of these regulations except that they presuppose that every insured person has the most intense interest in his insurance, and that whenever he removes, for instance, his first thought will be to notify his society or the Insurance Committee of his change of address. Experience has shown, however, that the very simplest requirements which necessitate writing or giving of notice to officials, who do not personally call at the houses of the industrial workers, are almost always ignored. When necessity arises for actual use to be made of the benefits provided the industrial worker is not slow to claim his rights. Such necessity when it arises is most frequently of an urgent character, and he is disposed to consider the delay, which is in reality due to his own default in not taking precautions earlier to see that everything was in order, not only as annoying but as unreasonable and vexatious. Unless, therefore, the regulations can be considerably simplified in regard to the duties imposed upon insured persons, or unless some inducement can be furnished to the secretaries and officials of the approved societies to inform members what to do and see that they do it, the panel system will not become really popular.

### Financial Provisions.

It is fortunate that highly trained officials only will have any occasion to master the intricacies of the financial regulations proposed to be adopted. The financial provisions are indeed the kernel of the new regulations, and they will demand patience and skill from all who essay the task of removing the shell. The medical profession and those who undertake to supply drugs and appliances have the right, however, to know the outlines of the system under which they are to receive remuneration. Briefly stated it is as follows: There is to be constituted a fund styled the " General Medical Benefit Fund " in the hands of the Insurance Commissioners. To this fund the appropriate contributions of the insured persons will be carried, together with any Parliamentary grant that may be made. This General Medical Benefit Fund is, as it were, the central reservoir, out of which will be paid to the Insurance Committees the sums required for medical treatment and attendance. These subdivided sums when they come into the hands of the Committees will be known as the Medical Benefit Fund.

The charges on the Medical Benefit Fund of each Committee will be met in the first place by dividing the amount at disposal among three funds called respectively the Panel Service Fund, the Institutions Fund, and the Special Arrangements Fund. The basis of apportionment is here the relative number of insured persons who have chosen the three respective ways of receiving benefit. We need not continue further the analysis of the two latter funds. The Panel Service Fund is, however, divided into two portions in the proportion of thirteen to four, the former being styled the Practitioners Fund and the latter the Drug Fund.

There are two principal ways in which the Practitioners Fund will actually be disposed of—namely, where the system of remuneration is (1) on a capitation basis, and (2) by attendance; though it is possible that in some districts the two methods may run concurrently, thus adding further complication. When payment is by attendance the system has from the commencement provided the means for the distribution of the whole sum available. This was not so in the case of the capitation system, and as this plan is by far the more general it will be well to quote as nearly as possible the actual words of the proposed regulation:

> Where the Committee have adopted a capitation system of payment, they shall credit to each practitioner on the panel, in respect of each of the persons included in his list at the commencement of each quarter, an amount calculated in accordance with the rate contained in his agreement with the Committee, and there shall be credited to such of the practitioners on the panel and in such proportions as are agreed between the Committee and the Panel Committee, or in default of agreement, as the Commissioners may determine, *such further capitation fees as are in the aggregate equal to the number of insured persons* (other than insured members of institutions and persons making their own arrangements) whose names are at the commencement of the quarter included in the Register of the Committee, and who have not at that date been accepted by or assigned to any practitioner on the panel, and in arriving at any such agreement the Committee shall have regard to the responsibility incurred by each practitioner on the panel to give treatment during that quarter to insured persons not included in his list at the commencement of the quarter.

It will be seen that we have printed in italics the words that are proposed to effect the solution of the disposal of the aggregate sum available. As no reference is made in the draft to the retrospective action of the regulations there is no solution, except by implication, as to the disposal of the surplus funds now in the hands of the Insurance Committees. It will be noticed that the regulations are directly contrary to the opinion of counsel recently given to the London Insurance Committee.

